# Association of *MMP3*, *MMP14*, and *MMP25* gene polymorphisms with cerebral stroke risk: a case-control study

**DOI:** 10.1186/s12920-023-01734-1

**Published:** 2023-11-20

**Authors:** Yanling Yin, Yu Zhang, Xiaobo Zhang, Qi Zhang, Jiachen Wang, Tian Yang, Chen Liang, Wu Li, Jie Liu, Xiaojuan Ma, Jinwei Duan, Wenzhen Shi, Ye Tian

**Affiliations:** 1 Department of Neurology, Xi’an Key Laboratory of Cardiovascular and Cerebrovascular Diseases, Xi’an No.3 Hospital, the Affiliated Hospital of Northwest University, Xi’an, Shaanxi 710018 China; 2 The College of Life Sciences, Northwest University, Xi’an, Shaanxi 710069 China; 3 Clinical Medical Research Center, Xi’an Key Laboratory of Cardiovascular and Cerebrovascular Diseases, Xi’an No.3 Hospital, the Affiliated Hospital of Northwest University, Xi’an, Shaanxi 710018 China

**Keywords:** Cerebral Stroke, Case-control study, *MMP3*, *MMP14*, *MMP25*, Single nucleotide polymorphisms

## Abstract

**Background:**

Cerebral stroke (CS) is the leading cause of death in China, and a complex disease caused by both alterable risk factors and genetic factors. This study intended to investigate the association of *MMP3*, *MMP14*, and *MMP25* single nucleotide polymorphisms (SNPs) with CS risk in a Chinese Han population.

**Methods:**

A total of 1,348 Han Chinese were recruited in this case-control study. Four candidate loci including rs520540 A/G and rs679620 T/C of *MMP3*, rs2236302 G/C of *MMP14*, and rs10431961 T/C of *MMP25* were successfully screened. The correlation between the four SNPs and CS risk was assessed by logistic regression analysis. The results were analyzed by false-positive report probability (FPRP) for chance or significance. The interactions between four SNPs associated with CS risk were assessed by multifactor dimensionality reduction (MDR).

**Results:**

rs520540 A/G and rs679620 C/T SNP in *MMP3* were associated with risk of CS in allele, codominant, dominant and log-additive models. Ischemic stroke risk were significantly lower in carriers with rs520540-A allele and rs679620-T allele than those with G/G or C/C genotypes. However, rs520540-A allele and rs679620-T allele were associated with higher risk of hemorrhagic stroke. Stratified analysis showed that these two SNPs were associated with reduced risk of CS in aged < 55 years, non-smoking and non-drinking participants, and rs679620 SNP also reduced CS risk in male participants. The levels of uric acid, high-density lipoprotein cholesterol, and eosinophil were different among patients with different genotypes of rs520540 and rs679620. No statistically significant association was found between *MMP14* rs2236302 G/C or *MMP25* rs10431961 T/C with CS even after stratification by stroke subtypes, age, gender as well as smoking and drinking conditions in all the genetic models.

**Conclusion:**

*MMP3* rs520540 A/G and rs679620 C/T polymorphisms were associated with CS risk in the Chinese Han population, which provides useful information for the prevention and diagnosis of CS.

**Supplementary Information:**

The online version contains supplementary material available at 10.1186/s12920-023-01734-1.

## Introduction

Cerebral stroke (CS), commonly known as “stroke”, also known as cerebrovascular accident, is an acute cerebrovascular disease caused by cerebral vascular obstruction or sudden rupture of blood vessels. CS includes ischemic stroke (IS) and hemorrhagic stroke (HS), of which IS (also known as cerebral infarction) is the most pervasive class of CS in clinic, accounting for about 70% ~ 80% of CS. HS is commonly caused by intracerebral hemorrhage (ICH) or subarachnoid hemorrhage. According to the Global Burden of Disease Study 2019, the overall incidence of CS in China was 39.9%, ranking first in the world [1]. CS has become the second most common cause of death and disability in the world [2], and the first cause of death in China [3]. CS is a complex disease caused by modifiable clinical risk factors (diet, physical inactivity, hypertension [4], smoking [5], diabetes [6], and dyslipidemia [7]) and genetic factors. The results of a meta-analysis of twin studies and family history studies have shown that there is a clear genetic predisposition to CS. Moreover, monozygotic twins are more likely to have CS at the same time than fraternal twins, and people with a family history of CS are more likely to suffer from CS [8]. Therefore, achieving a deep insight into the individual’s genetic makeup and clinical exposures will expand our understanding on the occurrence of CS.

Matrix metalloproteinase (MMP) is a kind of calcium ion-dependent endopeptidase that plays critical roles in the degradation of extracellular matrix (ECM) of blood vessels, thereby involving in the occurrence of many vascular diseases including cerebrovascular atherosclerosis, HS and IS [9]. The single nucleotide polymorphisms (SNPs) of *MMPs* are present since birth and are known to be associated with transcriptional activity, expression and changes in their enzymatic activities of MMPs [10]. Hence, we speculated that *MMPs* gene polymorphisms might be involved in the occurrence of CS. This case-control study selected four candidate SNPs (*MMP3*: rs520540 A/G, rs679620 T/C; *MMP14*: rs2236302 G/C; *MMP25*: rs10431961 T/C) in 1,348 Han Chinese and assessed the effect of these SNPs on the susceptibility to CS. Meanwhile, we further studied the association between SNPs and CS risk stratified by age, gender, smoking, and drinking. Our study will enrich the data of genetic loci related to CS risk in the Chinese Han population, and the discovered susceptibility loci can also be used as genetic markers for predicting CS risk and screening high-risk CS population, so as to provide theoretical basis for the early prevention and diagnosis of CS.

## Materials and methods

### Study participants

Study participants including 674 CS patients and 674 healthy individuals were recruited from Xi’an No.3 Hospital of Shaanxi Province. CS patients were newly diagnosed and confirmed by experienced specialists according to the diagnostic criteria based on computed tomography (CT) imaging. The inclusion criteria for cases were patients with a first or definite diagnosis of CS (cerebral infarction, cerebral hemorrhage, subarachnoid hemorrhage, and unclassified stroke) in an outpatient or inpatient setting. Patients with a history of other comorbid diseases (malignant tumors, metabolic disorders, central nervous system infections, etc.) and other genetic disorders were excluded. The control group included healthy people who underwent physical examination in the same hospital during the same period as case group and were not diagnosed with CS, and healthy individuals were matched with case group for age and sex (excluding confounding factors). Patients with complex chronic diseases (excluding patients with cancer or a history of cancer) were excluded. Besides, a professional physician conducted a baseline survey of all participants’ general demographic data (including gender, age, smoking and drinking status).

### Blood sample collection and DNA extraction

Admission peripheral blood samples from each participant were collected in EDTA-anticoagulated blood collection tubes and stored in a -20 °C refrigerator. After that, we extracted and purified DNA according to the kit instructions (GoldMag Co. Ltd., Xi’an, China). Eventually, the extracted DNA was stored in an ultra-low temperature freezer at -80 °C for future research.

### SNP selection and genotyping

A protein–protein interaction (PPI) network was conducted by STRING database (http://string-db.org) to explore the functional enrichment analysis of *MMP3*, *MMP14*, *MMP25*. Meanwhile, Kyoto Encyclopedia of Genes and Genomes (KEGG) [11, 12] and gene ontology (GO) [13–15] analysis was performed by R 4.0.5 software package. They were considered functionally important at the *p* < 0.05 level. Subsequently, SNPs were selected from the 1000 Genomes Project with minor allele frequency (MAF) > 0.05, Hardy-Weinberg equilibrium (HWE) > 0.05, and Tagger r^2^ < 0.8. Finally, this study selected two sites rs520540 A/G and rs679620 T/C on the *MMP3* gene, one site rs2236302 G/C on the *MMP14* gene, and one site rs10431961 T/C on the *MMP25* gene. The primers were designed by MassARRAY Assay Design software. All SNPs in this study were genotyped using the MassARRAY system (Agena, San Diego, CA, USA). SNP genotypes were generated using iPLEX chemistry. MALDI-TOF mass spectrometry was used to obtain profiles of different mass peaks of multiple reactions, and finally, genotyping was successfully completed.

### Statistical analysis

Demographic characteristics of the participants, including age (*t*-test), gender, smoking, and drinking (χ^2^ test) were tested by SPSS 21.0 (SPSS, Chicago, IL, USA) to determine whether all SNPs met HWE. Odds ratios (ORs) and 95% confidence intervals (CIs) were calculated by logistic regression models to evaluate the correlation between all candidate SNPs and CS risk. When OR < 1, it indicates that this SNP is a protective factor against CS; when OR = 1, it shows that this SNP has no effect on CS; when OR > 1, it indicates that this SNP is a risk factor for CS. A variety of genetic models were evaluated using PLINK 1.9 with wild-type alleles as references. All tests were two-sided, and *p* < 0.05 was considered statistically significant. Afterwards, the detection results were analyzed by false-positive report probability (FPRP) for chance or significance. Haplotype analysis was performed by Haploview to calculate linkage disequilibrium (LD). The interactions between four SNPs associated with CS risk were assessed by multifactor dimensionality reduction (MDR). Differences in clinical characteristics of patients with different genotypes were analyzed by one-way analysis of variance (ANOVA).

## Results

### Sample overview

Basic demographic and epidemiological information about subjects is presented in Table 1. There were 1,348 unrelated participants in this case-control study, including 674 CS patients (455 males (67.5%) and 219 females (32.5%)) with an average age of 54.85 ± 6.759 years, and 674 healthy individuals (443 males (65.5%) and 231 females (34.3%)) with an average age of 55.61 ± 9.130 years. There were no significant differences in mean age (*p* = 0.080), gender (*p* = 0.488), smoking (*p* = 0.827), and drinking (*p* = 0.827) between the two groups. However, there were significant discrepancies in the levels of uric acid (UA), high-density lipoprotein cholesterol (HDL-C), eosinophil (EOS), globulin (GLOB), monocyte (MON), low-density lipoprotein cholesterol (LDL-C), and red blood cell (RBC) between these two groups.

**Table 1:** Baseline characteristics of participants in cerebral stroke case and healthy control groups

Characteristics		Cases *N* = 674	Control *n* = 674	*p*
Age (years)	Mean ± SD	54.85 ± 6.759	55.61 ± 9.130	0.080
	> 55	377 (55.9%)	295 (43.8%)	
	≤ 55	297 (44.1%)	379 (56.2%)	
Gender	Male	455 (67.5%)	443 (65.5%)	0.488
	Female	219 (32.5%)	231 (34.3%)	
Smoking	Yes	319 (47.3%)	323 (47.9%)	0.827
	No	355 (52.7%)	351 (52.1%)	
Drinking	Yes	328 (48.7%)	332 (49.3%)	0.827
	No	346 (51.3%)	342 (50.7%)	
UA (µmol/L)		283.17 ± 89.14	322.45 ± 78.78	0.000*
HDL-C (mmol/L)		1.10 ± 0.24	1.15 ± 0.29	0.041*
EOS (%)		1.62 ± 1.72	2.28 ± 1.72	0.000*
GLOB (g/L)		25.31 ± 3.71	26.18 ± 3.28	0.001*
MON (%)		5.58 ± 2.57	7.02 ± 1.87	0.000*
LDL-C (mmol/L)		1.97 ± 0.68	2.67 ± 0.73	0.000*
RBC (×10^12^/L)		4.72 ± 0.66	4.87 ± 0.45	0.002*

UA, uric acid; HDL-C, high-density lipoprotein cholesterol; EOS, Eosinophil; GLOB, globulin; MON, monocytes; LDL-C, Low Density Lipoprotein; RBC, red blood cell

*p* < 0.05, bold text and ‘*’ represent statistical significance

#### Genotyping of candidate SNPs

**Fig. 1:**
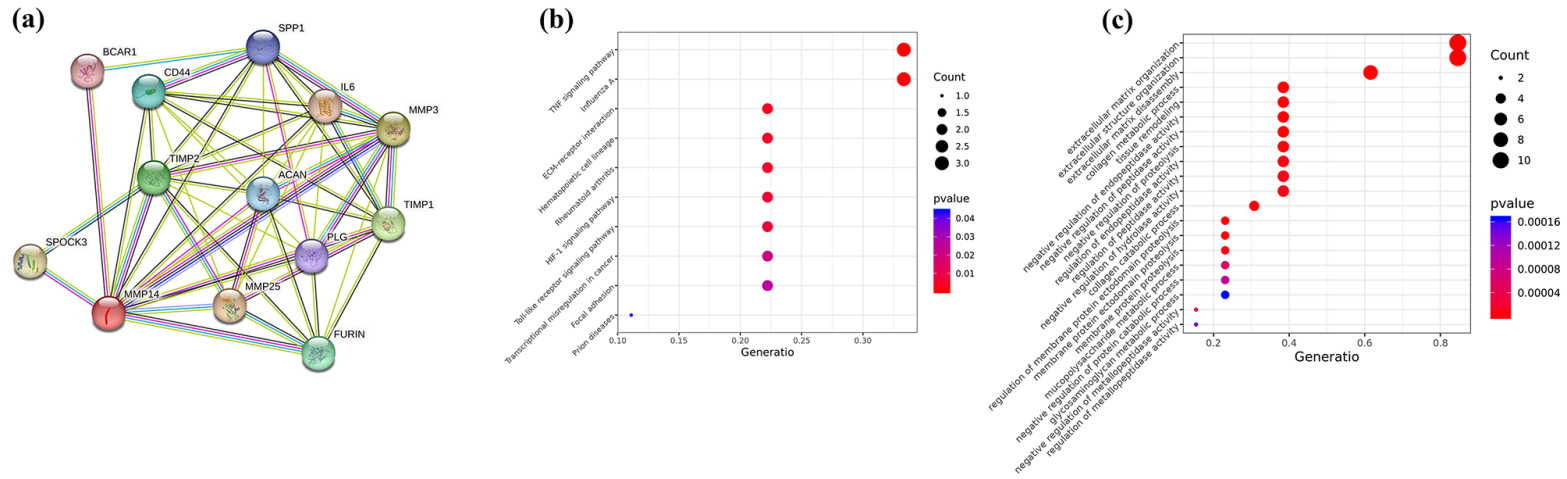
The functional enrichment analysis of *MMP3*, *MMP14* and *MMP25* genes. **(a)** The protein–protein interaction (PPI) network of *MMP3/MMP14/MMP25*. **(b)** The Kyoto Encyclopedia of Genes and Genomes (KEGG) enrichment analysis of genes. **(c)** The biological process (BP) of overlapping genes

**Fig. 2:**
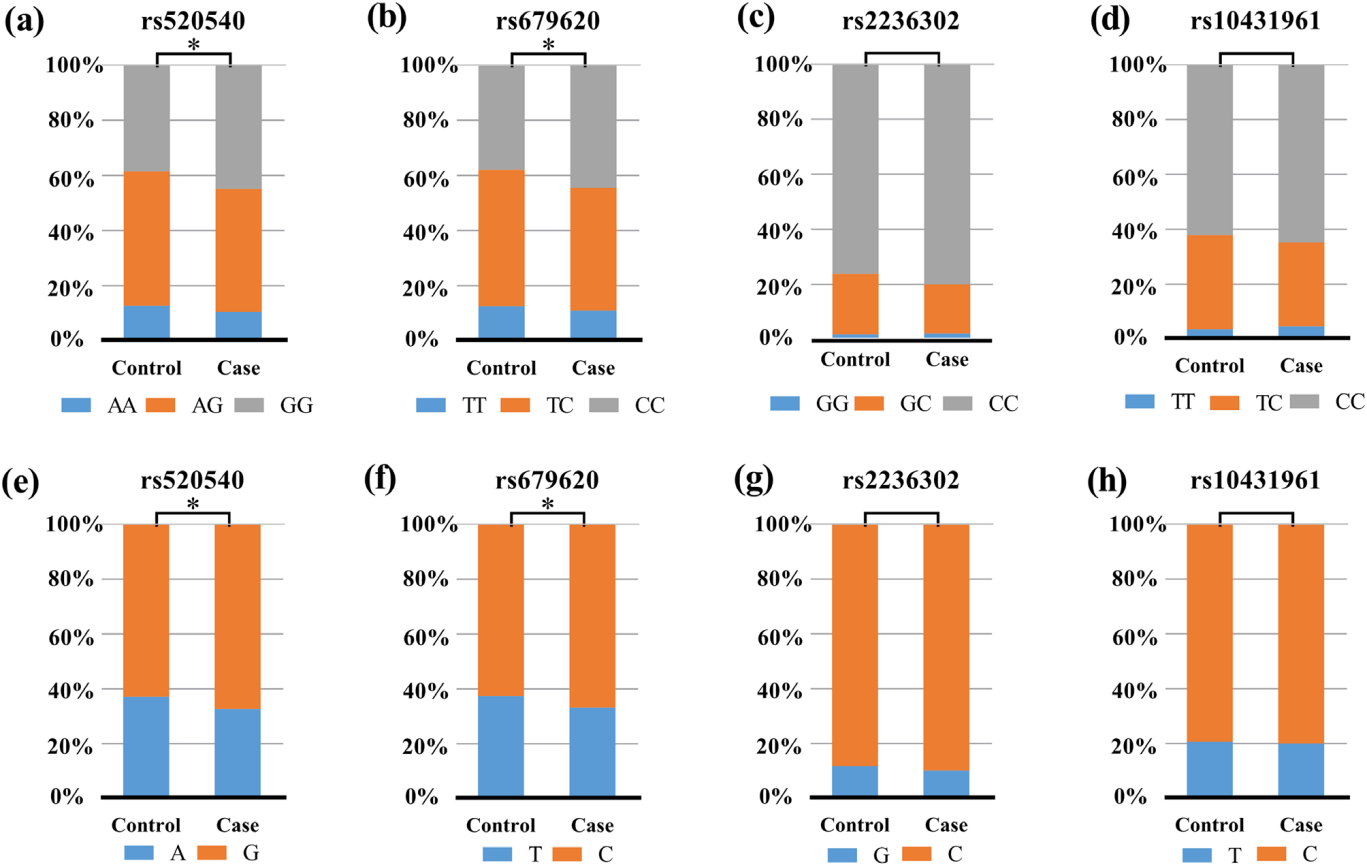
Comparisons of the genotype frequency and allele frequency of SNPs of *MMP3/MMP14/MMP25* genes in CS cases and healthy controls. Comparisons of the genotype frequency of rs520540 **(a)**, rs679620 **(b)** of *MMP3* gene, rs2236302 of *MMP14* **(c)** and rs10431961 of *MMP25***(d)** between the two groups. Comparisons of the allele frequency of rs520540 **(e)**, rs679620 **(f)** of *MMP3* gene, rs2236302 of *MMP14***(g)** and rs10431961 of *MMP25***(h)** between the two groups. ‘*’ indicate statistical significance at the 0.05 level

The results of the functional enrichment analysis are shown in Fig. 1. We constructed a PPI network analysis based on *MMP3/MMP14/MMP25* genes. The enrichment analysis showed that these genes were enriched in TNF signaling pathway. The possible biological process (BP) of these overlapping genes were related to the extracellular matrix organization and extracellular structure organization. In this study, four candidate loci were successfully screened: *MMP3* rs520540 A/G and rs679620 T/C, *MMP14* rs2236302 G/C, and *MMP25* rs10431961 T/C. These four SNPs were in line with HWE (*p* > 0.05), and MAF was greater than 5% in the tested population. The specific information about all candidate SNPs is shown in Table 2. In addition, we found significant differences in *MMP3* rs520540 A/G genotype frequency (*p* = 0.049) and allele frequency (*p* = 0.016) as well as *MMP3* rs679620 T/C genotype frequency (*p* = 0.033) and allele frequency (*p* = 0.012) between CS patients and healthy controls (Fig. 2).

**Table 2:** The basic information and HWE about the selected SNPs

Gene	SNP ID	Chrs	Position	Function	Alleles (A/B)	MAF		HWE (*p* value)
						Cases	Control	
*MMP3*	rs520540	11	102,838,694	Synonymous	A/G	0.327	0.371	0.284
*MMP3*	rs679620	11	102,842,889	Nonsynonymous	T/C	0.328	0.374	0.161
*MMP14*	rs2236302	14	22,843,345	Synonymous	G/C	0.101	0.119	0.268
*MMP25*	rs10431961	16	3,050,094	Synonymous	T/C	0.200	0.207	0.291

HWE, Hardy–Weinberg equilibrium; SNP, single nucleotide polymorphisms; Chrs, chromosome number; Alleles (A/B), minor/major allele; MAF, minor allele frequency

*p* > 0.05 indicates that the genotypes were in Hardy–Weinberg Equilibrium

### Association between candidate SNPs and CS risk

**Fig. 3:**
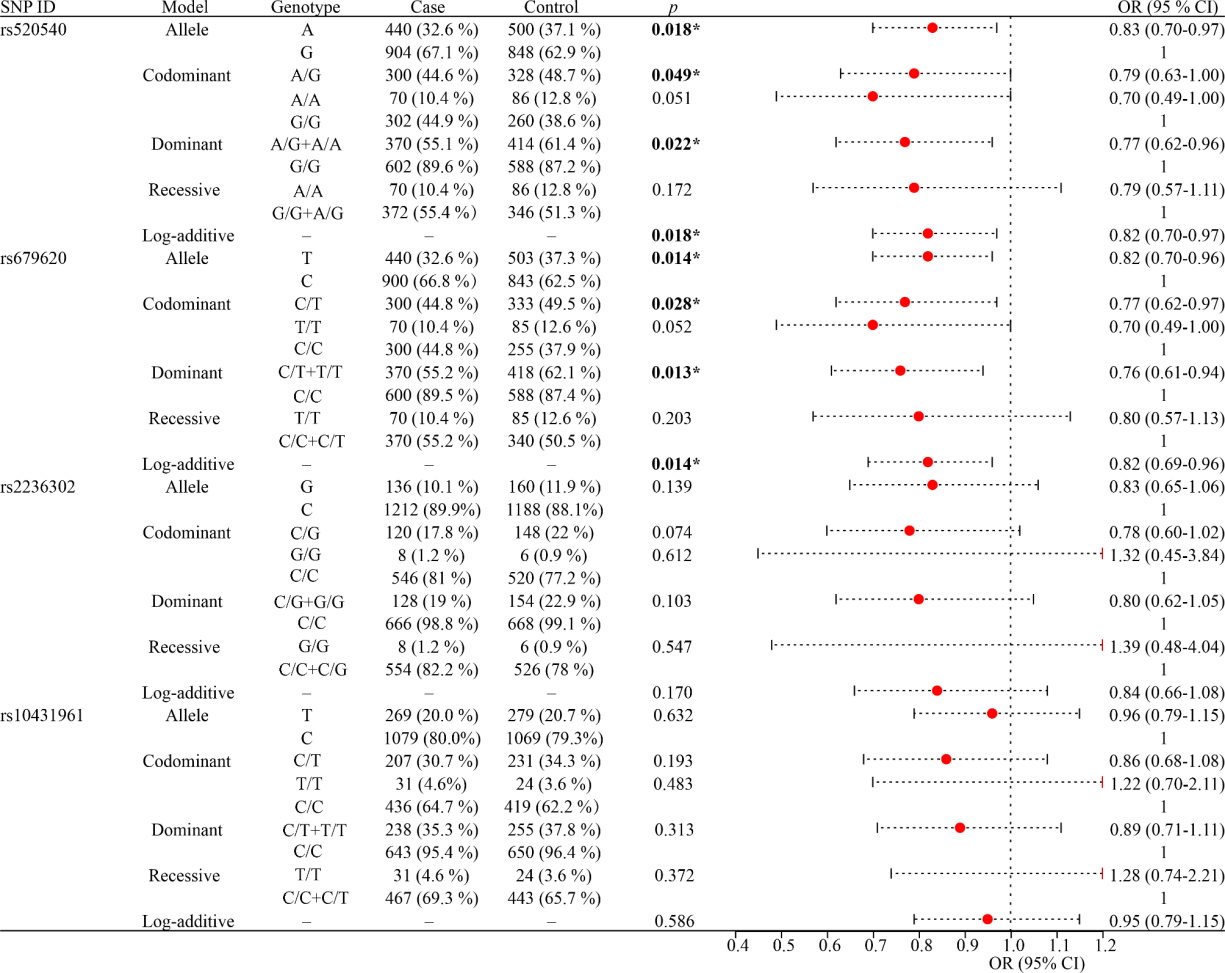
Analysis of the association between susceptibility of CS and SNPs. CS, cerebral stroke; SNP, single nucleotide polymorphisms; OR, odds ratio; CI, confidence interval; *p* values represent adjusted for age, gender, smoking, and drinking; *p* < 0.05, bold text and ‘*’ indicate statistical significance

The association between the the four candidate SNPs and the risk of CS is shown in Fig. 3. The results showed that *MMP3* rs520540 A/G and rs679620 T/C SNPs were significantly associated with CS risk. In particular, rs520540 A/G significantly reduced CS risk under the allele (A vs. G: OR = 0.83, 95% CI 0.70–0.97, *p* = 0.018), codominant (AG vs. GG: OR = 0.79, 95% CI 0.63-1.00, *p* = 0.049), dominant (AG-AA vs. GG: OR = 0.77, 95% CI 0.62–0.96, *p* = 0.022), and log-additive models (OR = 0.82, 95% CI 0.70–0.97, *p* = 0.018). Meanwhile, rs679620 T/C could notably decrease the risk of CS under the allele (T vs. C: OR = 0.82, 95% CI 0.70–0.96, *p* = 0.014), codominant (CT vs. CC: OR = 0.77, 95% CI 0.62–0.97, *p* = 0.028), dominant (CT-TT vs. CC: OR = 0.76, 95% CI 0.61–0.94, *p* = 0.013), and log-additive models (OR = 0.82, 95% CI 0.69–0.96, *p* = 0.014). However, no correlation was found between the remaining two SNPs (*MMP14* rs2236302 G/C, and *MMP25* rs10431961 T/C) and CS risk (*p* > 0.05).

Furthermore, we stratified the CS patients into ischemic stroke and hemorrhagic stroke groups, and analyzed the association between these four SNPs with stroke subtypes. In this study, 610 patients suffered from IS and 64 patients had HS, and the healthy controls were randomly selected using SPSS 26.0 software at 1:1 of case-control for the two types of stroke, respectively. After adjusting for age, gender, smoking and drinking history, the OR with 95% CI and *p* values were calculated between SNPs and IS/HS risk using logistic analysis (Table 3). The results showed that *MMP3* rs520540 A allele and rs679620 T allele were associated with decreased risk of IS (A vs. G: OR = 0.64, 95% CI 0.07–0.85, *p* < 0.001; T vs. C: OR = 0.80, 95% CI 0.07–0.84, *p* = 0.018), their protective effects against IS were also found in codominant, dominant and log-additive models. Meanwhile, *MMP3* rs520540 A allele and rs679620 T allele may associate with higher risk of HS, although no significant difference were indicated. The *MMP3* rs520540 A/G genotype and rs679620 C/T genotype carried a significant increase in the risk of HS, particularly in dominant and log-additive models. Furthermore, no statistically significant association was found between *MMP14* rs2236302 G/C or *MMP25* rs10431961 T/C and two stroke subtypes.

**Table 3:** Logistic regression analysis of the association between MMP3, MMP14 and MMP25 polymorphism with susceptibility of ischemic or hemorrhagic stroke

SNP ID	Model	Genotype	Ischemic stroke				Hemorrhagic stroke			
			Case	Control	OR (95% CI)^a^	*p* ^*a*^	Case	Control	OR (95% CI)^a^	*p* ^*a*^
rs520540	Allele	A	586 (48.1%)	454 (37.2%)	0.64 (0.07, 0.85)	< 0.001*	52 (40.6%)	47 (36.7%)	1.18 (0.79, 1.56)	0.522
		G	632 (51.9%)	766 (62.8%)	1.00		76 (59.4%)	81 (63.3%)	1.00	
	Codominant	A/G	64 (10.5%)	294 (48.2%)	0.66 (0.49,0.88)	0.005*	40 (62.5%)	25 (39.1%)	14.84 (1.68, 130.83)	0.015*
		A/A	261 (42.8%)	80 (13.1%)	0.57 (0.35,0.92)	0.021*	6 (9.4%)	11 (17.2%)	3.46 (0.18, 66.82)	0.411
		G/G	284 (46.6%)	236 (38.7%)	1.00		18 (28.1%)	28 (43.8%)	1.00	
	Dominant	A/G + A/A	325 (53.4%)	374 (61.3%)	0.64 (0.48, 0.85)	0.002*	46 (71.9%)	28 (43.8%)	11.82 (1.37,101.80)	0.025*
		G/G	284 (46.6%)	236 (37.7%)	1.00		18 (28.1%)	36 (56.2%)		
	Recessive	A/A	64 (10.5%)	80 (13.1%)	0.71 (0.46, 1.12)	0.140	6 (9.4%)	11 (17.2%)	0.49 (0.05, 4.66)	0.535
		G/G + A/G	545 (89.5%)	530 (86.9%)	1.00		58(90.6%)	53 (82.8%)		
	Log-additive	–			0.73 (0.55, 0.97)	0.029*			0.11 (0.02, 0.55)	0.008*
rs679620	Allele	T	391 (32.1%)	447 (37.2%)	0.80 (0.07, 0.84)	0.018*	52 (40.6%)	49 (38.3%)	1.10 (1.00, 1.46)	0.709
		C	827 (67.9%)	753 (62.8%)	1.00		76 (59.4%)	79 (61.7%)	1.00	
	Codominant	C/T	261 (42.8%)	289 (48.9%)	1.64 (1.02, 2.63)	0.041*	40 (62.5%)	25 (39.1%)	4.19 (0.42, 42.30)	0.224
		T/T	65 (10.7%)	79 (13.0%)	1.04 (0.65, 1.66)	0.879	6 (9.4%)	12 (18.8%)	0.30 (0.02, 5.78)	0.422
		C/C	283 (46.4%)	232 (38.0%)	1.00		18 (28.1%)	27 (42.2%)	1.00	
	Dominant	C/T + T/T	326 (53.5%)	377 (61.9%)	0.62 (0.47, 0.82)	0.001*	46 (71.9%)	37 (52.8%)	11.23 (1.28, 98.20)	0.029*
		C/C	283 (46.5%)	232 (38.1%)	1.00		18 (28.1%)	27 (47.2%)	1.00	
	Recessive	T/T	65 (10.7%)	79 (13.0%)	0.76 (0.50, 1.21)	0.262	6 (9.4%)	12 (18.8%)	0.49 (0.05, 4.66)	0.535
		C/C + C/T	544 (89.3%)	530 (87.0%)			58 (90.6%)	52 (81.2%)	1.00	
	Log-additive	–			0.69 (0.52, 0.92)	0.010*			0.11 (0.02, 0.59)	0.010*
rs2236302	Allele	G	127 (10.4%)	143 (11.7%)	0.88 (0.35, 1.29)	0.302	9 (7.0%)	12 (9.4%)	0.73 (0.52, 1.00)	0.494
		C	1093 (89.6%)	1077 (88.3)	1.00		119 (93.0%)	116 (90.6%)	1.00	
	Codominant	C/G	113 (18.5%)	135 (22.1%)	2.27 (0.16, 33.07)	0.548	7 (10.9%)	12 (18.8%)	3.388 (0.36, 31.96)	0.286
		G/G	7 (1.1%)	4 (0.7%)	2.73 (0.19, 39.23)	0.460	1 (1.6%)	0 (0)	--	--
		C/C	490 (80.3%)	471 (77.2%)	1.00		56 (87.5%)	52 (81.2%)		
	Dominant	C/G-G/G	120 (19.7%)	139 (22.8%)	0.82 (0.58, 1.16)	0.269	8 (12.5%)	12 (18.8%)	0.30 (0.031, 2.78)	0.286
		C/C	490 (80.3%)	471 (77.2%)	1.00		56 (87.5%)	52 (81.2%)	1.00	
	Recessive	G/G	7 (1.1%)	4 (0.7%)	0.38 (0.03, 5.43)	0.476	1 (1.6%)	0 (0.0)	--	--
		C/C-C/G	603 (98.9%)	606 (99.3%)	1.00		63 (98.4%)	64 (100.0%)	1.00	
	Log-additive	–			1.20 (0.85, 1.70)	0.311			3.39 (0.36, 31.96)	0.286
rs10431961	Allele	T	250 (20.5%)	254 (20.8%)	0.98 (0.75, 1.42)	0.842	19 (14.8%)	28 (21.9%)	0.62 (0.25, 1.64)	0.146
		C	970 (79.5%)	966 (79.2%)	1.00		109 (85.2%)	100 (78.1%)	1.00	
	Codominant	C/T	194 (31.8%)	206 (33.8%)	0.86 (0.43, 1.73)	0.680	13 (20.3%)	26 (40.7%)	0.17 (0.02, 1.76)	0.137
		T/T	28 (4.6%)	24 (3.9%)	0.81 (0.39, 1.65)	0.557	3 (4.7%)	1(1.6%)	0.43 (0.04, 4.33)	0.476
		C/C	388 (63.6%)	380 (62.3%)	1.00		48 (75.0%)	37(57.8%)	1.00	
	Dominant	C/T + T/T	222 (36.4%)	230 (37.7%)	0.96 (0.72, 1.28)	0.769	16 (25.0%)	27 (42.2%)	0.34 (0.07, 1.78)	0.202
		C/C	388 (63.6%)	380 (62.3%)	1.00		48 (75.0%)	37 (57.8%)	1.00	
	Recessive	T/T	28 (4.6%)	24 (3.9%)	1.18 (0.60, 2.36)	0.630	3 (4.7%)	1 (1.6%)	0.00 (0.00, INF)	1.000
		C/C + C/T	582 (95.4%)	586 (96.1%)	1.00		61 (95.3%)	63 (98.4%)	1.00	
	Log-additive	–			1.08 (0.80, 1.45)	0.608			2.52 (0.48, 13.34)	0.277

SNP, Single nucleotide polymorphisms; OR, odds ratio; CI, Confidence interval;

*p* < 0.05, bold text and ‘*’ indicate statistical significance

^a^Adjusted for age, sex, smoking and dinking

### Stratified analysis of the association between candidate SNPs and CS risk

**Age and gender** Stratified by age and gender, the correlation between the four SNPs and CS risk is presented in Table 4. The results indicated that rs520540 could remarkably decrease the risk of CS in subjects aged ≤ 55 years under a variety of genetic models (allele: OR = 0.79, 95% CI 0.63–0.99, *p* = 0.039; codominant: OR = 0.66, 95% CI 0.47–0.92, *p* = 0.014; dominant: OR = 0.67, 95% CI 0.49–0.91, *p* = 0.011; log-additive: OR = 0.77, 95% CI 0.61–0.97, *p* = 0.030). rs679620 was a protective factor not only in CS patients aged ≤ 55 years under multiple genetic models (allele: OR = 0.80, 95% CI 0.63-1.00, *p* = 0.048; codominant: OR = 0.63, 95% CI 0.45–0.88, *p* = 0.007; dominant: OR = 0.65, 95% CI 0.48–0.89, *p* = 0.008; log-additive: OR = 0.78, 95% CI 0.62–0.99, *p* = 0.038), but also in CS patients aged > 55 years under two genetic models (codominant: OR = 0.58, 95% CI 0.35–0.98, *p* = 0.040; recessive: OR = 0.62, 95% CI 0.38-1.00, *p* = 0.049). Meanwhile, it was also a protective factor in males (dominant: OR = 0.76, 95% CI 0.58–0.99, *p* = 0.045; log-additive: OR = 0.81, 95% CI 0.67–0.99, *p* = 0.044). Additionally, rs2236302 could notably reduce CS risk in males (allele: OR = 0.72, 95% CI 0.53–0.99, *p* = 0.039; codominant: OR = 0.69, 95% CI 0.49–0.97, *p* = 0.035). Nevertheless, we did not find any association of rs10431961 with CS risk and any association between the four candidate SNPs and CS risk in females (*p* > 0.05).

**Table 4:** The SNPs associated with susceptibility of cerebral stroke in the subgroup tests (age and gender)

SNP ID	Model	Genotype	Age				Gender			
			OR (95% CI)	*p*	OR (95% CI)	*p*	OR (95% CI)	*p*	OR (95% CI)	*p*
			≤ 55 (*N* = 676)		> 55 (*N* = 672)		Male (*N* = 898)		Female (*N* = 450)	
rs520540	Allele	A	0.79 (0.63–0.99)	0.039*	0.86 (0.68–1.08)	0.185	0.84 (0.69–1.02)	0.086	0.80 (0.61–1.05)	0.104
		G	1.00		1.00		1.00		1.00	
	Codominant	A/G	0.66 (0.47–0.92)	0.014*	0.92 (0.66–1.28)	0.637	0.83 (0.62–1.10)	0.200	0.68 (0.45–1.02)	0.064
		A/A	0.69 (0.41–1.15)	0.156	0.63 (0.38–1.05)	0.074	0.69 (0.44–1.07)	0.097	0.68 (0.35–1.31)	0.245
		G/G	1.00		1.00		1.00		1.00	
	Dominant	A/G + A/A	0.67 (0.49–0.91)	0.011*	0.86 (0.63–1.17)	0.329	0.80 (0.61–1.05)	0.101	0.68 (0.46–1.01)	0.054
		G/G	1.00		1.00		1.00		1.00	
	Recessive	A/A	0.85 (0.53–1.39)	0.524	0.66 (0.41–1.06)	0.084	0.76 (0.50–1.15)	0.189	0.85 (0.46–1.57)	0.601
		G/G + A/G	1.00		1.00		1.00		1.00	
	Log-additive	–	0.77 (0.61–0.97)	0.030*	0.83 (0.66–1.05)	0.119	0.83 (0.68–1.01)	0.066	0.77 (0.57–1.04)	0.089
rs679620	Allele	T	0.80 (0.63-1.00)	0.048*	0.83 (0.67–1.04)	0.111	0.82 (0.68-1.00)	0.053	0.81 (0.62–1.07)	0.137
		C	1.00		1.00		1.00		1.00	
	Codominant	C/T	0.63 (0.45–0.88)	0.007*	0.90 (0.65–1.26)	0.552	0.78 (0.58–1.03)	0.082	0.73 (0.48–1.10)	0.129
		T/T	0.75 (0.45–1.25)	0.264	0.58 (0.35–0.98)	0.040*	0.69 (0.44–1.08)	0.102	0.68 (0.35–1.30)	0.240
		C/C	1.00		1.00		1.00		1.00	
	Dominant	C/T + T/T	0.65 (0.48–0.89)	0.008*	0.83 (0.61–1.14)	0.247	0.76 (0.58–0.99)	0.045*	0.72 (0.48–1.07)	0.100
		C/C	1.00		1.00		1.00		1.00	
	Recessive	T/T	0.95 (0.58–1.54)	0.825	0.62 (0.38-1.00)	0.049*	0.79 (0.52–1.20)	0.261	0.81 (0.44–1.50)	0.504
		C/C + C/T	1.00		1.00		1.00		1.00	
	Log-additive	–	0.78 (0.62–0.99)	0.038*	0.81 (0.64–1.02)	0.069	0.81 (0.67–0.99)	0.044*	0.79 (0.59–1.06)	0.120
rs2236302	Allele	G	0.74 (0.51–1.05)	0.093	0.90 (0.65–1.27)	0.558	0.72 (0.53–0.99)	0.039*	1.06 (0.72–1.58)	0.763
		C	1.00		1.00		1.00		1.00	
	Codominant	C/G	0.81 (0.54–1.21)	0.298	0.77 (0.53–1.13)	0.184	0.69 (0.49–0.97)	0.035*	1.10 (0.69–1.74)	0.687
		G/G	0.57 (0.11–3.02)	0.506	4.36 (0.52–36.90)	0.176	1.69 (0.40–7.14)	0.479	0.73 (0.14–3.87)	0.712
		C/C	1.00		1.00		1.00		1.00	
	Dominant	C/G-G/G	0.79 (0.54–1.18)	0.252	0.83 (0.57–1.20)	0.312	0.72 (0.51–1.01)	0.055	1.07 (0.68–1.68)	0.756
		C/C	1.00		1.00		1.00		1.00	
	Recessive	G/G	0.59 (0.11–3.15)	0.538	4.62 (0.55-39.00)	0.160	1.81 (0.43–7.68)	0.418	0.72 (0.14–3.78)	0.694
		C/C-C/G	1.00		1.00		1.00		1.00	
	Log-additive	–	0.80 (0.55–1.15)	0.228	0.90 (0.64–1.27)	0.553	0.77 (0.56–1.06)	0.109	1.04 (0.69–1.57)	0.850
rs10431961	Allele	T	0.98 (0.75–1.29)	0.911	0.93 (0.71–1.21)	0.570	0.95 (0.76–1.20)	0.684	0.95 (0.68–1.33)	0.772
		C	1.00		1.00		1.00		1.00	
	Codominant	C/T	0.84 (0.60–1.18)	0.312	0.86 (0.62–1.20)	0.380	0.91 (0.68–1.21)	0.525	0.74 (0.49–1.13)	0.163
		T/T	1.47 (0.67–3.22)	0.34	1.19 (0.53–2.64)	0.671	0.99 (0.50–1.93)	0.966	2.24 (0.78–6.46)	0.134
		C/C	1.00		1.00		1.00		1.00	
	Dominant	C/T + T/T	0.90 (0.65–1.24)	0.509	0.89 (0.65–1.23)	0.486	0.92 (0.70–1.21)	0.550	0.84 (0.56–1.25)	0.382
		C/C	1.00		1.00		1.00		1.00	
	Recessive	T/T	1.55 (0.71–3.37)	0.271	1.25 (0.57–2.76)	0.578	1.02 (0.52–1.98)	0.959	2.46 (0.86–7.02)	0.093
		C/C + C/T	1.00		1.00		1.00		1.00	
	Log-additive	–	0.98 (0.74–1.28)	0.861	0.95 (0.72–1.24)	0.690	0.94 (0.75–1.19)	0.625	0.97 (0.69–1.37)	0.869

SNP, Single nucleotide polymorphisms; OR, odds ratio; CI, Confidence interval;

*p* < 0.05, bold text and ‘*’ indicate statistical significance

**Smoking and drinking** Stratified by smoking and drinking, the correlation between the four SNPs and CS risk is presented in Table 5. The results indicated that rs520540 reduced the risk of CS in both non-smokers (OR = 0.79, 95% CI 0.42–0.98, *p* = 0.034) and non-drinkers (OR = 0.78, 95% CI 0.62–0.97, *p* = 0.024) participants in the allele model. And rs679620 reduced the risk of CS in non-smoking subjects in the allele model (OR = 0.77, 95% CI 0.62–0.96, *p* = 0.018). Meanwhile, it decreased CS risk in non-drinking participants under a variety of genetic models (allele: OR = 0.76, 95% CI 0.61–0.95, *p* = 0.014; codominant: OR = 0.59, 95% CI 0.36–0.99, *p* = 0.047; dominant: OR = 0.72, 95% CI 0.52–0.98, *p* = 0.039; log-additive: OR = 0.76, 95% CI 0.61–0.97, *p* = 0.025). Furthermore, we found no correlation of rs2236302 and rs10431961 with CS risk and no association between the four candidate SNPs and CS risk in smokers and drinkers (*p* > 0.05).

**Table 5:** The SNPs associated with susceptibility of cerebral stroke in the subgroup tests (smoking and drinking)

SNP ID	Model	Genotype	Smoking				Drinking			
			OR (95% CI)	*p*	OR (95% CI)	*p*	OR (95% CI)	*p*	OR (95% CI)	*p*
			Yes (*N* = 642)		No (*N* = 706)		Yes (*N* = 660)		No (*N* = 688)	
rs520540	Allele	A	0.87 (0.69–1.09)	0.224	0.79 (0.64–0.98)	0.034*	0.88 (0.70–1.11)	0.279	0.78 (0.62–0.97)	0.024*
		G	1.00		1.00		1.00		1.00	
	Codominant	A/G	0.77 (0.55–1.07)	0.122	0.84 (0.61–1.16)	0.287	0.78 (0.56–1.09)	0.144	0.79 (0.57–1.10)	0.201
		A/A	0.85 (0.49–1.46)	0.550	0.66 (0.41–1.08)	0.101	0.85 (0.50–1.43)	0.535	0.61 (0.37–1.02)	0.830
		G/G	1.00		1.00		1.00		1.00	
	Dominant	A/G + A/A	0.78 (0.57–1.08)	0.131	0.80 (0.59–1.09)	0.151	0.79 (0.58–1.09)	0.151	0.75 (0.55–1.03)	0.216
		G/G	1.00		1.00		1.00		1.00	
	Recessive	A/A	0.97 (0.58–1.63)	0.911	0.73 (0.46–1.15)	0.177	0.96 (0.58–1.58)	0.873	0.69 (0.43–1.12)	0.970
		G/G + A/G	1.00		1.00		1.00		1.00	
	Log-additive	–	0.86 (0.68–1.10)	0.233	0.82 (0.66–1.03)	0.087	0.87 (0.69–1.10)	0.253	0.78 (0.62–0.99)	0.290
rs679620	Allele	T	0.88 (0.70–1.11)	0.273	0.77 (0.62–0.96)	0.018*	0.89 (0.71–1.12)	0.316	0.76 (0.61–0.95)	0.014*
		C	1.00		1.00		1.00		1.00	
	Codominant	C/T	0.77 (0.55–1.08)	0.133	0.81 (0.58–1.12)	0.195	0.80 (0.57–1.12)	0.187	0.75 (0.54–1.05)	0.091
		T/T	0.90 (0.51–1.56)	0.697	0.63 (0.39–1.03)	0.067	0.86 (0.51–1.46)	0.569	0.59 (0.36–0.99)	0.047*
		C/C	1.00		1.00		1.00		1.00	
	Dominant	C/T + T/T	0.79 (0.57–1.09)	0.159	0.77 (0.56–1.04)	0.090	0.81 (0.59–1.11)	0.193	0.72 (0.52–0.98)	0.039*
		C/C	1.00		1.00		1.00		1.00	
	Recessive	T/T	1.03 (0.61–1.74)	0.922	0.71 (0.45–1.12)	0.143	0.96 (0.58–1.59)	0.883	0.69 (0.43–1.12)	0.137
		C/C + C/T	1.00		1.00		1.00		1.00	
	Log-additive	–	0.88 (0.69–1.12)	0.305	0.80 (0.64-1.00)	0.051	0.88 (0.70–1.12)	0.298	0.76 (0.61–0.97)	0.025*
rs2236302	Allele	G	0.90 (0.63–1.27)	0.533	0.78 (0.55–1.09)	0.146	0.90 (0.65–1.26)	0.553	0.76 (0.53–1.09)	0.129
		C	1.00		1.00		1.00		1.00	
	Codominant	C/G	0.92 (0.62–1.38)	0.702	0.72 (0.49–1.05)	0.085	0.80 (0.55–1.18)	0.266	0.84 (0.56–1.25)	0.380
		G/G	0.90 (0.25–3.24)	0.872	2.20 (0.22–22.18)	0.504	2.01 (0.49–8.27)	0.332	0.54 (0.09–3.46)	0.519
		C/C	1.00		1.00		1.00		1.00	
	Dominant	C/G-G/G	0.92 (0.63–1.36)	0.686	0.74 (0.50–1.07)	0.111	0.85 (0.59–1.23)	0.393	0.82 (0.55–1.22)	0.331
		C/C	1.00		1.00		1.00		1.00	
	Recessive	G/G	0.91 (0.25–3.29)	0.891	2.36 (0.23–23.74)	0.467	2.11 (0.52–8.65)	0.299	0.56 (0.09–3.58)	0.543
		C/C-C/G	1.00		1.00		1.00		1.00	
	Log-additive	–	0.93 (0.66–1.32)	0.688	0.77 (0.54–1.11)	0.163	0.92 (0.65–1.29)	0.611	0.82 (0.56–1.19)	0.297
rs10431961	Allele	T	0.93 (0.71–1.22)	0.604	0.98 (0.76–1.26)	0.861	1.05 (0.81–1.37)	0.701	0.87 (0.66–1.13)	0.292
		C	1.00		1.00		1.00		1.00	
	Codominant	C/T	0.89 (0.63–1.24)	0.481	0.85 (0.61–1.19)	0.343	0.97 (0.69–1.35)	0.836	0.80 (0.57–1.12)	0.201
		T/T	1.18 (0.45–3.12)	0.737	1.11 (0.55–2.22)	0.775	1.51 (0.66–3.45)	0.323	0.92 (0.42–2.01)	0.830
		C/C	1.00		1.00		1.00		1.00	
	Dominant	C/T + T/T	0.91 (0.65–1.26)	0.554	0.88 (0.65–1.21)	0.444	1.01 (0.73–1.40)	0.939	0.82 (0.59–1.13)	0.216
		C/C	1.00		1.00		1.00		1.00	
	Recessive	T/T	1.23 (0.47–3.24)	0.672	1.17 (0.59–2.32)	0.661	1.53 (0.68–3.47)	0.304	0.99 (0.45–2.14)	0.970
		C/C + C/T	1.00		1.00		1.00		1.00	
	Log-additive	–	0.94 (0.70–1.26)	0.692	0.94 (0.73–1.22)	0.645	1.06 (0.81–1.40)	0.672	0.86 (0.66–1.13)	0.290

SNP, Single nucleotide polymorphisms; OR, odds ratio; CI, confidence interval;

*p* < 0.05, bold text and ‘*’ indicate statistical significance

### Differences in clinical characteristics of patients with different genotypes

The associations between the four candidate SNPs and clinical characteristics of CS patients with different genotypes are presented in Table 6. The results showed that the rs520540 AA genotype was linked with the reduced levels of UA, HDL-C, and EOS in CS patients. And the rs679620 TT genotype was connected with the increased levels of UA, HDL-C, EOS, and GLOB in CS patients. The *MMP14* rs2236302 GG genotype was associated with the reduced levels of MON. The *MMP25* rs10431961 TT genotype was associated with the increased levels of LDL-C and the reduced levels of RBC.

**Table 6:** Comparisons of laboratory parameters at admission in CS patients (*N* = 674) carrying various genotypes of selected SNPs

	UA (µmol/L)	HDL-C(mmol/L)	EOS (%)	GLOB (g/L)	MON (%)	LDL-C(mmol/L)	RBC (×10^12^/L)
rs520540							
A/A	289.39 ± 88.89	1.00 ± 0.26	1.90 ± 1.66	25.50 ± 4.20	5.62 ± 2.19	1.91 ± 0.82	4.75 ± 0.66
A/G	263.92 ± 83.72	1.15 ± 0.25	1.19 ± 1.19	24.94 ± 3.98	5.66 ± 2.64	2.04 ± 0.65	4.66 ± 0.62
G/G	305.47 ± 91.80	1.07 ± 0.22	2.04 ± 2.14	25.70 ± 3.16	5.50 ± 2.64	1.90 ± 0.67	4.78 ± 0.71
*p*	0.013*	0.006*	0.003*	0.510	0.923	0.355	0.636
rs679620							
T/T	292.97 ± 90.55	1.01 ± 0.26	1.90 ± 1.71	25.47 ± 4.22	5.62 ± 2.23	1.91 ± 0.84	4.76 ± 0.68
C/T	265.75 ± 82.50	1.15 ± 0.25	1.24 ± 1.25	25.04 ± 3.89	5.61 ± 2.63	2.06 ± 0.64	4.67 ± 0.62
C/C	304.77 ± 92.38	1.07 ± 0.22	1.99 ± 2.09	25.73 ± 3.14	5.54 ± 2.64	1.87 ± 0.67	4.78 ± 0.71
*p*	0.007*	0.025*	0.014*	0.044*	0.982	0.272	0.577
rs2236302							
G/G	287.86 ± 83.04	1.13 ± 0.21	2.30 ± 0.97	26.36 ± 1.85	4.58 ± 3.42	2.22 ± 1.01	5.07 ± 0.49
C/G	297.53 ± 96.10	1.04 ± 0.23	1.67 ± 1.62	25.79 ± 3.60	4.68 ± 2.61	1.92 ± 0.56	4.61 ± 0.84
C/C	280.69 ± 88.45	1.11 ± 0.25	1.59 ± 1.76	25.18 ± 3.78	5.81 ± 2.48	1.96 ± 0.69	4.73 ± 0.62
*p*	0.626	0.384	0.552	0.517	0.045*	0.585	0.231
rs10431961							
T/T	316.75 ± 137.70	1.00 ± 0.16	1.41 ± 1.65	25.56 ± 3.11	6.65 ± 1.94	1.83 ± 0.53	4.94 ± 0.73
C/T	289.15 ± 89.24	1.06 ± 0.25	1.63 ± 1.74	25.56 ± 4.01	5.41 ± 2.41	1.76 ± 0.47	4.86 ± 0.52
C/C	277.47 ± 83.96	1.13 ± 0.24	1.63 ± 1.72	25.17 ± 3.63	5.58 ± 2.69	2.08 ± 0.76	4.64 ± 0.70
*p*	0.274	0.063	0.913	0.757	0.366	0.005*	0.031*

UA, uric acid; HDL-C, high-density lipoprotein cholesterol; EOS, Eosinophil; GLOB, globulin; MON, monocytes; LDL-C, Low Density Lipoprotein; RBC, red blood cell

*p* < 0.05, bold text and ‘*’ represent statistical significance

### FPRP analysis

Detailed results of FPRP analysis are displayed in Table S1. The results showed that the correlation between rs679620 and CS risk in subjects aged > 55 years was not noteworthy at a prior probability level of 0.25 and a FPRP threshold of 0.2. Meanwhile, the association between rs679620 and CS risk in non-drinkers under the homozygous model was not noteworthy. The FPRP values of other results were all less than 0.2 at a prior probability level of 0.25, suggesting that these positive results were noteworthy.

### LD and haplotype analysis

**Fig. 4:**
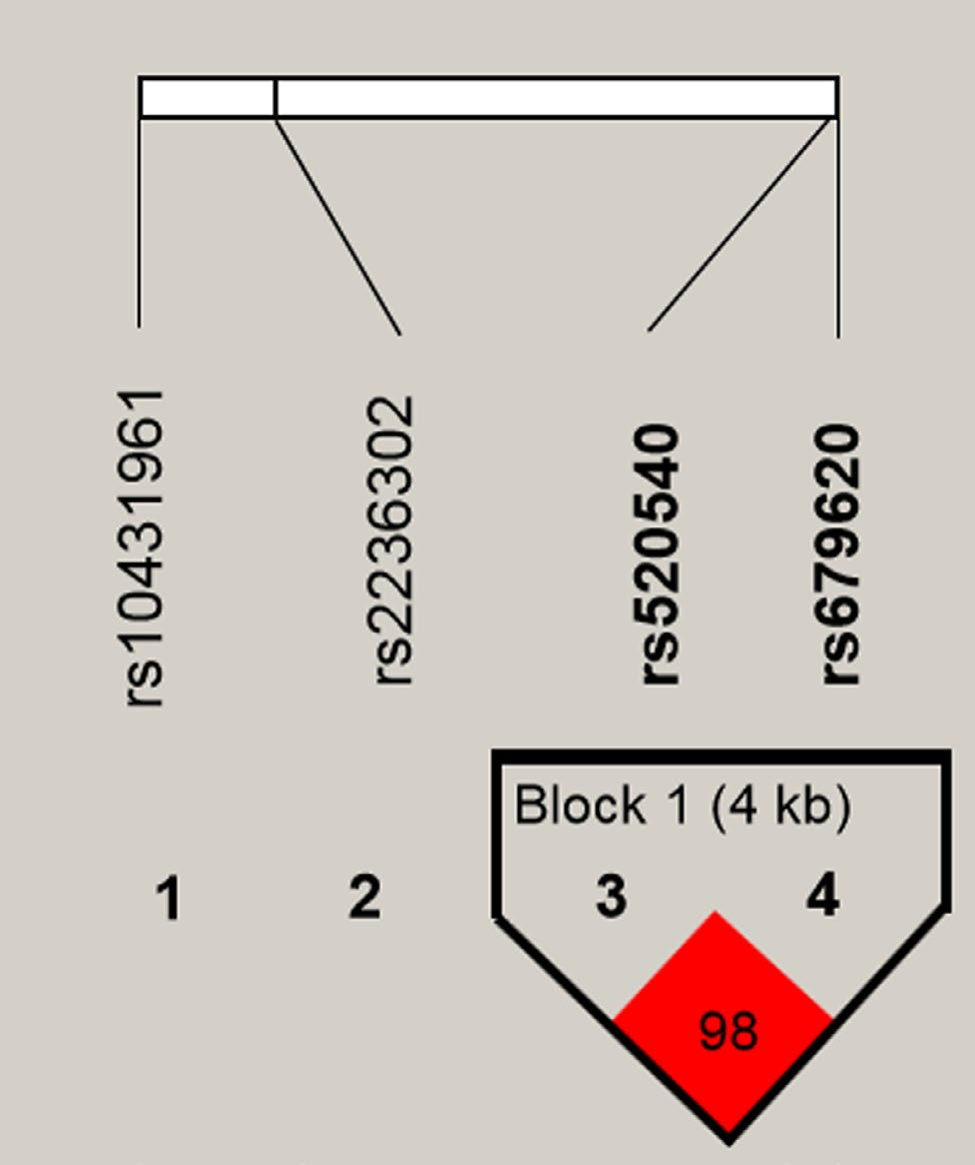
Linkage disequilibrium (LD) plots containing four polymorphisms

LD results of the candidate SNPs are shown in Fig. 4, and haplotype analysis results are presented in Table S2. The results indicated that there was linkage disequilibrium between rs520540 and rs679620. Meanwhile, the results of haplotype analysis showed that the distribution of the haplotype A_rs520540_T_rs679620_ was associated with a decreased risk of CS (OR = 0.82, *p* = 0.018).

### MDR analysis

**Fig. 5:**
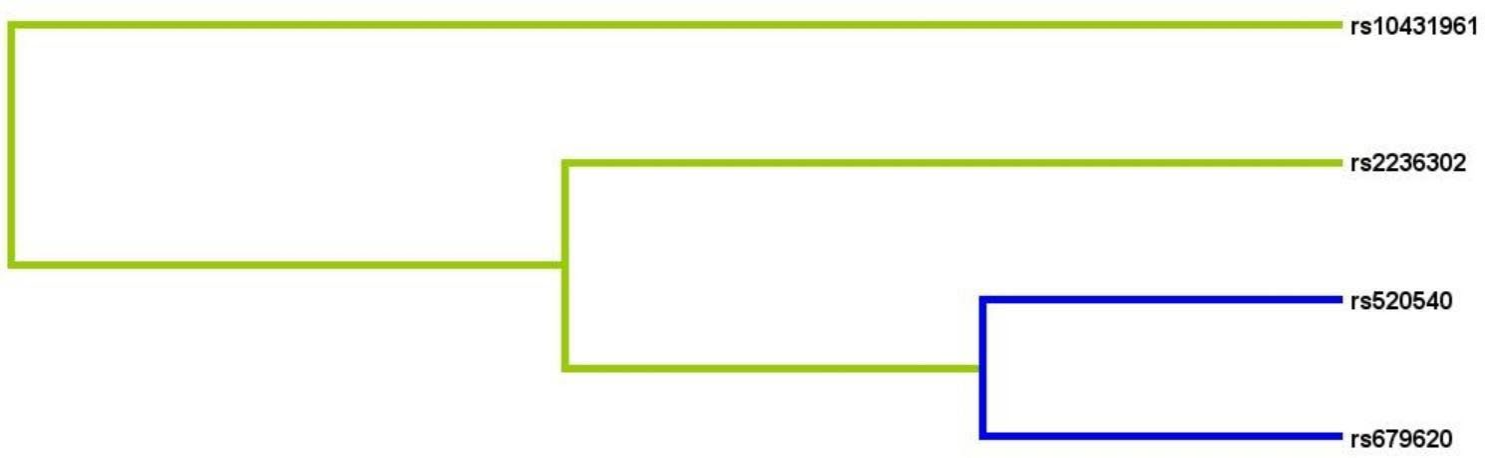
Dendrogram of SNP-SNP interactions

MDR analysis of SNP-SNP interactions is shown in Fig. 5. The blue lines represent that SNPs have redundant effects in modulating CS risk. Details of SNP-SNP interactions are presented in Table 7. The results showed that the best prediction model was the rs679620 single-site model (good CVC: 9/10, the largest testing balanced accuracy: 0.520, *p* = 0.0080).

**Table 7:** SNP–SNP interaction models analyzed by the MDR method

Model	Training Bal. Acc	Testing Bal. Acc	OR (95% CI)	*p* value	CVC
rs679620	0.536	0.520	1.34 (1.08–1.67)	**0.0080**	9/10
rs679620, rs10431961	0.541	0.508	1.48 (1.16–1.89)	**0.0016**	4/10
rs679620, rs2236302, rs10431961	0.549	0.503	1.47 (1.19–1.83)	**0.0005**	9/10
rs679620, rs520540, rs2236302, rs10431961	0.553	0.502	1.52 (1.22–1.90)	**0.0002**	10/10

MDR, multifactor dimensionality reduction; Bal. Acc., balanced accuracy; CVC, cross-validation consistency; OR, odds ratio; 95% CI, 95% confidence interval;

*p* values were calculated using χ^2^ tests; *p* < 0.05 and bold text indicate statistical significance

## Discussion

This study is the first to analyze the association of *MMP3* (rs520540 A/G and rs679620 T/C), *MMP14* (rs2236302 G/C), and *MMP25* (rs10431961 T/C) gene polymorphisms with CS risk in Chinese Han population. We found that *MMP3* gene polymorphisms (rs520540 A/G and rs679620 T/C) were associated with CS risk. Notably, they played a protective role against IS development, rather than in HS.

ECM remodeling is an essential process in the pathogenesis of atherosclerosis and IS. MMPs act as fundamental mediators for matrix turnover by their protein-digesting enzymatic activities in catalyzing the breakdown of major ECM components, such as collagen, elastin, gelatin, and other glycoproteins and proteoglycans [16]. MMP3 is an important member of MMPs that activates other MMPs, such as MMP1, MMP7, and MMP9, making MMP3 essential in connective tissue and ECM remodeling, resulting in plaque instability [17, 18]. MMP3 is expressed in various vascular tissues and cells, including endothelial cells and vascular smooth muscle cells, thereby modulating various vascular diseases. Alvarez-sabín et al. [19] have found that the expression of *MMP3* is up-regulated in patients with cerebral hemorrhage. A study in a mouse middle cerebral artery embolization model has revealed that tissue-type plasminogen activator (t-PA) treatment induces *MMP3* expression levels in peripheral blood and increases hemorrhagic events, which is thought to be related to the disruption of vascular endothelial barrier by MMP3 [20]. *MMP3* SNP is associated with reduced in vitro promoter activity and expression [21]. *MMP3* (-1171) 5 A/6A + 5 A/5A genotypes and 5 A allele were significantly higher in patients with IS than in controls and contributed to different subtypes of IS susceptibility [16]. Circulating MMP3 level was significantly higher in patients with 5 A/6A or 5 A/5A genotype than in patients with 6 A/6A genotype. *MMP3* 5 A allele carrier may bear a higher risk of recurrence among patients with the stroke subtype of large-artery atherosclerosis [22]. Kaplan et al. found that *MMP3* rs3025058 SNP was associated with increased risk of hemorrhagic stroke [23]. In the overall analysis, this study found that two SNPs of *MMP3* (rs520540 A/G and rs679620 T/C) were associated with the reduced risk of IS in the Chinese Han population. In contrary to our findings, the study by Kim et al. [24] has shown that both rs520540 and rs679620 of *MMP3* increase the risk of IS in the Korean population. Some studies have shown that *MMP* gene polymorphisms can be influenced by racial and ethnic background and ultimately affect the onset of IS [10]. Therefore, we speculate that racial differences may cause the inconsistency between our results and those of previous studies, and we need to further verify our hypothesis in different populations. At the same time, the results demonstrated that rs520540-A allele and rs679620-T allele were associated with higher risk of hemorrhagic stroke. Our finding that the same *MMP3* haplotype (rs520540-A/G allele and rs679620-T/C) appeared to protect against thrombotic events (i.e., ischemic stroke) and promote bleeding events (i.e., hemorrhagic stroke) may be explained by cleavage of coagulation and fibrinolytic proteins (plasminogen, plasminogen activator inhibitor-1, fibrinogen) by MMP3.

Former research has revealed that risk factors such as age, gender [25], and smoking [5] contribute to the development of CS. Consequently, our study stratified the participants according to the above factors. The results indicated that both rs520540 and rs679620 of *MMP3* significantly reduced CS risk in non-smoking and non-drinking participants. Consistent with our results, the study by Larsson et al. [5] has shown that some gene polymorphisms is positively and statistically associated with IS, large artery stroke, and small vessel stroke in smokers. Another study has shown that there is a correlation between drinking and an increased risk of CS [26]. Thus, the association of rs520540 and rs679620 with the risk of CS may be affected by smoking and drinking. Meanwhile, we noticed rs679620 also reduced the risk of CS in male participants. Some studies have shown that genetic polymorphisms are correlated with CS risk in males [27, 28], which is consistent with our findings, whereas some studies have demonstrated that genetic polymorphisms have a significant correlation with CS risk in females [29, 30]. Therefore, we consider there may be gender differences in susceptibility to CS and further validation is needed to support our conclusions.

Tumor necrosis factor α (TNFα) is mainly secreted by microglia and plays an important role in maintaining homeostasis in the central nervous system [31]. Simultaneously, TNFα is a proinflammatory cytokine related to innate immune response, which is related to a variety of neurodegenerative diseases including Parkinson’s disease, Alzheimer’s disease, multiple sclerosis, ischemic brain injury, etc., and is considered to be one of the important indicators of the severity of IS [32, 33]. TNF modulates tissue damage in experimental CS and is a potential target for future CS therapy [34]. However, excessive TNFα are associated with inflammatory cascade reactions by amplifying and exacerbating the inflammatory response and, in severe cases, triggering systemic inflammatory response syndrome [35, 36]. Therefore, appropriate TNF plays an important proinflammatory role in CS. Our study found that the most important functional terms enriched in *MMP3/MMP14* genes was TNF signaling pathway. And the possible BP of *MMP3/MMP14/MMP25* genes were related to the extracellular matrix organization and extracellular structure organization. TNF signaling pathway has been shown to induce the expression of MMPs [37]. In addition, TNFα can activate nuclear factor of kappaB (NF-κB), and the activated NF-κB further induces the secretion of TNFα, which leads to the increased release of MMP activity and finally promotes cell invasion and metastasis [38]. Therefore, we hypothesized that *MMP3* rs520540/rs679620 and *MMP14* rs2236302 may play a role in the occurrence of CS through TNF signaling pathway. However, further experiments are needed to verify our conjecture.

In addition, studies have shown that the levels of UA, HDL-C and EOS in CS patients are significantly higher than those in normal people [39–41]. In our study, we found that the rs520540 A/G genotype was linked with the reduced levels of UA and EOS and higher HDL-C in CS patients. And this pattern was true for *MMP3* rs679620 T/C in affecting the levels of UA, EOS and HDL-C. Therefore, rs520540 A/G and rs679620 T/C seemed exhibiting protective roles against CS.

However, our study still has certain limitations. First, it was limited by the relative sample size in elucidating the association between SNPs and the occurance of stroke, particularly for the hemorrhagic stroke. Second, all participants were recruited in one medical center and only Han Chinese population were enrolled, thus the conclusion should be extrapolated with caution since genetic variation strongly related to ethnic background. Meanwhile, the conclusion should be validated in more clinical centers in a large sample size. Third, our study associated the *MMP3* SNPs with laboratory parameters, and deep insights into the association between SNPs and IS etiological subtypes, severity and prognosis should be explored in further studies due to the disease heterogeneity. Besides, the interplay between *MMP3* SNP and that in other *MMPs* members should be comprehensively analyzed depending complete sequencing and studies on the underlying mechanism are needed. Despite the above shortcoming, our study sheds new light on the association of *MMP3* (rs520540 A/G and rs679620 T/C), *MMP14* (rs2236302 G/C), and *MMP25* (rs10431961 T/C) gene polymorphisms with CS risk.

## Conclusion

In summary, this study first explored the correlation of *MMP3* (rs520540 A/G and rs679620 T/C), *MMP14* (rs2236302 G/C), and *MMP25* (rs10431961 T/C) gene polymorphisms with CS risk in the Chinese Han population. The results showed that there was a certain association between *MMP3* gene polymorphisms (rs520540 A/G and rs679620 T/C) and CS risk. *MMP3* rs520540 A/G and rs679620 T/C may exhibit different paradigms in affecting the initiation and progression of IS and HS, where rs520540 A and rs679620 T alleles exhibited protective roles against IS while promoted the occurrence of HS. Our study further enriches the data of susceptibility loci for CS in the Chinese Han population, and provides a preliminary molecular basis for the prevention, diagnosis and treatment of CS.

### Electronic supplementary material

Below is the link to the electronic supplementary material.

Supplementary Material 1: **Table S1** The FPRP and statistical power values of the positive results in this study. **Table S2** Haplotype frequencies and the association with the risk of cerebral stroke.

## Data Availability

The datasets generated and/or analysed during the current study are available in the [figshare] repository, 10.6084/m9.figshare.21769499.v1.
