# Supplementary material for: Association of MMP3, MMP14, and MMP25 gene polymorphisms with cerebral stroke risk: a case-control study
Source: BMC Med Genomics. 2023 Nov 20;16:297. doi: 10.1186/s12920-023-01734-1 (PMC10659043; doi:10.1186/s12920-023-01734-1)
Supplement: Supplementary file 1 — Supplementary Material 1: Table S1 The FPRP and statistical power values of the positive results in this study. Table S2 Haplotype frequencies and the association with the risk of cerebral stroke. [file 12920_2023_1734_MOESM1_ESM.docx]

Table S1 The FPRP and statistical power values of the positive results in this study.

| SNP ID | Model | Genotype | OR (95 % CI) | *p* | Statistical Power^a^ (%) | Prior probability | | |
| --- | --- | --- | --- | --- | --- | --- | --- | --- |
|  |  |  |  |  |  | 0.25 | 0.1 | 0.01 |
| **Overall analysis** |  |  |  |  |  |  |  |  |
| rs520540 | Allele | A/G | 0.83 (0.70-0.97) | 0.018 | 99.7 | 0.054^b^ | 0.147 ^b^ | 0.655 |
|  | Genotype | AG/GG | 0.79 (0.63-1.00) | 0.049 | 92.1 | 0.140 ^b^ | 0.328 | 0.843 |
|  | Dominant | AG-AA/GG | 0.77 (0.62-0.96) | 0.022 | 90.0 | 0.063 ^b^ | 0.168 ^b^ | 0.690 |
|  | Log-additive | – | 0.82 (0.70-0.97) | 0.018 | 99.2 | 0.059 ^b^ | 0.157 ^b^ | 0.673 |
| rs679620 | Allele | T/C | 0.82 (0.70-0.96) | 0.014 | 99.5 | 0.039 ^b^ | 0.110 ^b^ | 0.575 |
|  | Genotype | CT/CC | 0.77 (0.62-0.97) | 0.028 | 88.9 | 0.082 ^b^ | 0.212 | 0.747 |
|  | Dominant | CT-TT/CC | 0.76 (0.61-0.94) | 0.013 | 88.7 | 0.037 ^b^ | 0.104 ^b^ | 0.560 |
|  | Log-additive | – | 0.82 (0.69-0.96) | 0.014 | 99.5 | 0.039 ^b^ | 0.110 ^b^ | 0.575 |
| **Subgroup analysis: age (age ≤ 55）** |  |  |  |  |  |  |  |  |
| rs520540 | Allele | A/G | 0.79 (0.63-0.99) | 0.039 | 93.0 | 0.116 ^b^ | 0.282 | 0.812 |
|  | Genotype | AG/GG | 0.66 (0.47-0.92) | 0.014 | 47.6 | 0.082 ^b^ | 0.212 | 0.747 |
|  | Dominant | AG-AA/GG | 0.67 (0.49-0.91) | 0.011 | 51.3 | 0.057 ^b^ | 0.154 ^b^ | 0.667 |
|  | Log-additive | – | 0.77 (0.61-0.97) | 0.030 | 88.9 | 0.082 ^b^ | 0.212 | 0.747 |
| rs679620 | Allele | T/C | 0.80 (0.63-1.00) | 0.048 | 94.5 | 0.137 ^b^ | 0.322 | 0.840 |
|  | Genotype | CT/CC | 0.63 (0.45-0.88) | 0.007 | 37.0 | 0.052 ^b^ | 0.141 ^b^ | 0.643 |
|  | Dominant | CT-TT/CC | 0.65 (0.48-0.89) | 0.008 | 43.7 | 0.047 ^b^ | 0.129 ^b^ | 0.620 |
|  | Log-additive | – | 0.78 (0.62-0.99) | 0.038 | 90.2 | 0.120 ^b^ | 0.291 | 0.819 |
| **Subgroup analysis: age (age > 55）** |  |  |  |  |  |  |  |  |
| rs679620 | Genotype | TT/CC | 0.58 (0.35-0.98) | 0.040 | 30.1 | 0.294 | 0.555 | 0.932 |
|  | Recessive | TT/CC | 0.62 (0.38-1.00) | 0.049 | 38.3 | 0.281 | 0.540 | 0.928 |
| **Subgroup analysis: gender (male）** |  |  |  |  |  |  |  |  |
| rs679620 | Dominant | CT-TT/CC | 0.76 (0.58-0.99) | 0.045 | 82.5 | 0.154 ^b^ | 0.353 | 0.857 |
|  | Log-additive | – | 0.81 (0.67-0.99) | 0.044 | 97.1 | 0.109 ^b^ | 0.268 | 0.801 |
| rs2236302 | Allele | G/C | 0.72 (0.53-0.99) | 0.039 | 68.2 | 0.160 ^b^ | 0.363 | 0.862 |
|  | Genotype | CG/CC | 0.69 (0.49-0.97) | 0.035 | 57.8 | 0.145 ^b^ | 0.337 | 0.849 |
| **Subgroup analysis: smoking (no）** |  |  |  |  |  |  |  |  |
| rs520540 | Allele | A/G | 0.79 (0.64-0.98) | 0.034 | 93.9 | 0.093 ^b^ | 0.235 | 0.772 |
| rs679620 | Allele | T/C | 0.77 (0.62-0.96) | 0.018 | 90.0 | 0.063 ^b^ | 0.168 ^b^ | 0.690 |
| **Subgroup analysis: drinking (no）** |  |  |  |  |  |  |  |  |
| rs520540 | Allele | A/G | 0.78 (0.62-0.97) | 0.024 | 92.1 | 0.077 ^b^ | 0.199 ^b^ | 0.733 |
| rs679620 | Allele | T/C | 0.76 (0.61-0.95) | 0.014 | 87.5 | 0.052 ^b^ | 0.141 ^b^ | 0.643 |
|  | Genotype | TT/CC | 0.59 (0.36-0.99) | 0.047 | 32.2 | 0.299 | 0.561 | 0.934 |
|  | Dominant | CT-TT/CC | 0.72 (0.52-0.98) | 0.039 | 68.8 | 0.138 ^b^ | 0.325 | 0.841 |
|  | Log-additive | – | 0.76 (0.61-0.97) | 0.025 | 85.4 | 0.088 ^b^ | 0.225 | 0.761 |

FPRP, false-positive report probability; SNP, single nucleotide polymorphism; OR, Odds ratio; CI, Confidence interval.

^a^ Statistical power^a^ was calculated using the number of the OR and *p* values in this table.

^b^ The level of false-positive report probability threshold was set at 0.2, and noteworthy findings are presented.

Table S2 Haplotype frequencies and the association with the risk of cerebral stroke.

| Chr | Gene | SNP | Haplotype | Frequency | | Adjusted | |
| --- | --- | --- | --- | --- | --- | --- | --- |
|  |  |  |  | Cases | Control | OR (95 % CI) | *p* |
| 11 | *MMP3* | rs520540\|rs679620 | GC | 0.669 | 0.623 | 1.00 | – |
| 11 | *MMP3* | rs520540\|rs679620 | AT | 0.325 | 0.368 | 0.82 (0.70-0.97) | **0.018** |

Chrs, chromosome number; SNP, single nucleotide polymorphism;

Adjusted, logistic regression models were used to adjust for age + gender + smoking + drinking;

OR, Odds ratio; CI, Confidence interval.

*p* < 0.05 and bold text indicates statistical significance.
